# Supplementary material for: Neuronal growth on high-aspect-ratio diamond nanopillar arrays for biosensing applications
Source: Sci Rep. 2023 Apr 11;13:5909. doi: 10.1038/s41598-023-32235-x (PMC10090193; doi:10.1038/s41598-023-32235-x)
Supplement: Supplementary file 1 — Supplementary Information. [file 41598_2023_32235_MOESM1_ESM.pdf]

Supplementary Material

Histograms for the different pitches

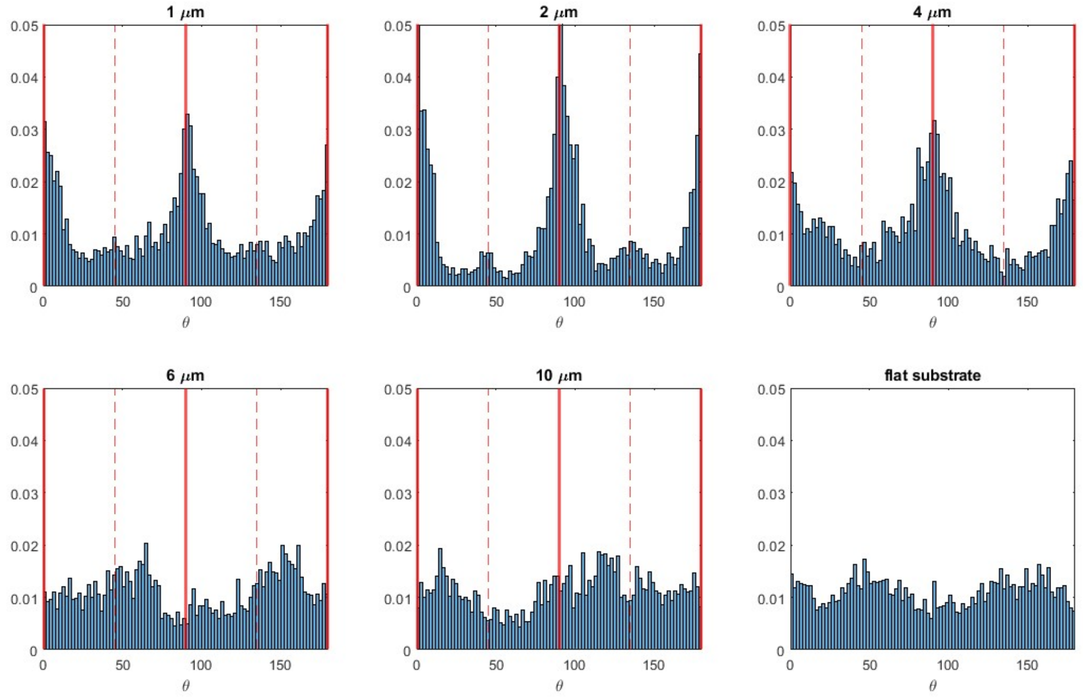

Figure S1. Histograms reporting the axon direction with respect to one of the main axes of the grid, for all the pitches present in our sample design. The detail of the analysis can be found in the main text. Red lines mark the two perpendicular directions along nearest neighbors of the array. Red dashed lines correspond to 45° and 135°. Each histogram is obtained analysing a number of SEM images between 10 and 15.

Preliminary results on neuronal culture on diamond substrate

We report here the results from the first test we did about culturing neurons on diamond. As reported in literature, a thin functionalization layer is typically necessary to promote adhesion between the cells and the diamonds. In particular we tried:

- poly-l-lysine (Bio-Techne, 3438-100)
- laminin (SIGMA-ALDRICH, L2020)
- poly-l-lysine (Bio-Techne, 3438-100) and laminin (SIGMA-ALDRICH, L2020)

Only the combination of the two was successful, while using just one of the coating resulted in poor quality of the culture (visible already from optical microscope inspection).

For the final experiment we plated at a density of 150’000 cells/ml and we imaged the neurons after 10 days, with the results reported in the main text. Preliminary attempts considered higher density (2x) and longer incubation time, up to 15 days. In these cases, we observed that it was not possible to see the diamond nanostructures, since they were completely embedded in the culture. An example of this situation is reported in Fig. S2.

Effect of the functionalization layer on the ODMR measurements

In this section we describe the preliminary experiment performed to evaluate the effects of the functionalization layer on the ODMR measurements. On the one hand, the functionalization layer is expected to potentially induce some effects only on the NV centers in proximity of the surface, on the other hand, we are currently using a diamond with a uniform distribution of NV centers in all the volume. Therefore, we focus our analysis on the thinnest pillars, having a diameter of 100 nm. In this way,

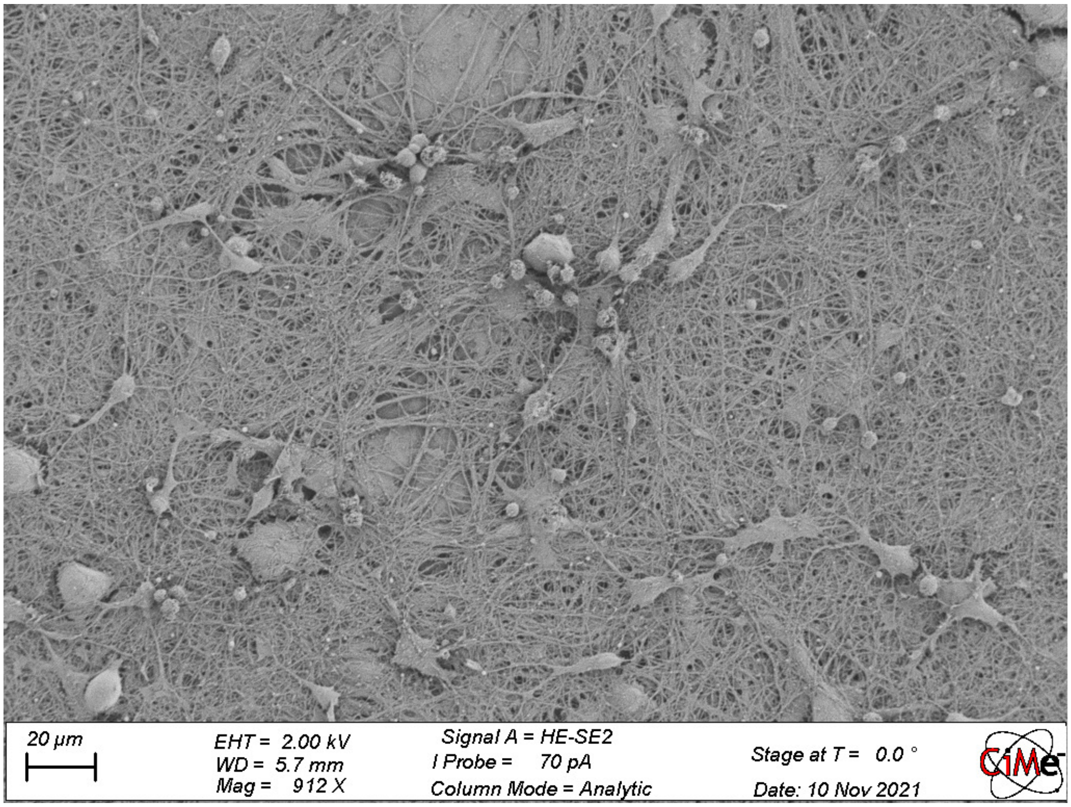

Figure S2. SEM image of primary hippocampal neuron plated on nanostructured diamond, after 14 days of incubation and with higher cell concentration with respect to what was chosen for the final experiment.

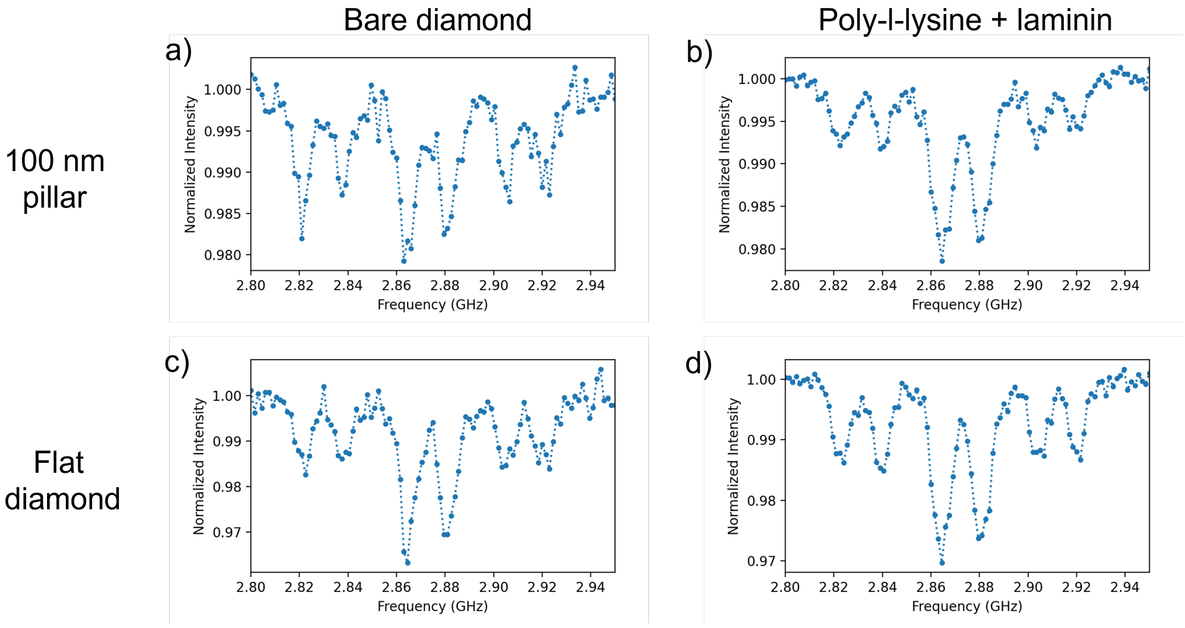

Figure S3. ODMR spectra from a) 100 nm pillars, bare diamond; b) 100 nm pillars, with poly-l-lysine and laminin coating; c) flat diamond, bare diamond; d) flat diamond, with poly-l-lysine and laminin coating.

all the NV centers in the excitation volume have a maximum distance of 50 nm from the surface, allowing to detect potential detrimental effects of the functionalization layer.

Using our confocal set-up, we acquire ODMR spectra from 100 nm pillars, with and without coating, maintaining all the other parameters (microwave power, optical excitation power, bias magnetic field) constant. The coating is performed exactly as it was done for plating the cells.

In Fig. S3 the results are reported. Also ODMR spectra from flat diamond are acquired as comparison. We can conclude that the coatings do not seem to have a major influence on the ODMR contrast. It is only slightly reduced, by the same amount in the bulk and in pillars. Therefore, we could say that the sensitivity will be only slightly degraded by the coating, without compromising the sensing protocols applicability.

Resting membrane potential

In the following table we report the average cell membrane resting potentials measured in the different tested arrays.

Resting membrane potential (mV)	$p(\mu\text{m}) \times d(\text{nm})$
-55	4×300
-60	1×500
-61	1×400
-49	10×100
-49	6×200
-63	2×200
-60	flat

On the amplitude of EPSPs recordings

As mentioned in the text the amplitude of the EPSPs signal we recorded (10 mV) is higher compared to typical values reported in literature. We here discuss better this point, suggesting some hypothesis explaining the phenomenon.

First of all, we need to take into account that the neurons are not in physiological conditions, and their neurites are not receiving the same signals for connecting than in physiology. It is possible that anomalous (in this case more than usual) connectivity happens between 2 neurons. It is possible that, due to the conditions of the culture (smaller neurite growth and higher interconnectivity), the amplitude of EPSP is observed larger than the value typically recorded for neurons in the physiology of brain tissue. Also, for the cell corresponding to the measurement reported in the figure, we were injecting -40pA to keep the recording at -65mV. Injection of negative current might have an amplifying effect on the amplitude of EPSPs.
